# New Developments of RNAi in *Paracoccidioides brasiliensis*: Prospects for High-Throughput, Genome-Wide, Functional Genomics

**DOI:** 10.1371/journal.pntd.0003173

**Published:** 2014-10-02

**Authors:** Tercio Goes, Elisa Flavia L. C. Bailão, Cristiane R. Correa, Adriana Bozzi, Luara I. Santos, Dawidson A. Gomes, Celia M. A. Soares, Alfredo M. Goes

**Affiliations:** 1 Departamento de Bioquímica e Imunologia, Instituto de Ciências Biológicas, Universidade Federal de Minas Gerais (UFMG), Belo Horizonte, Minas Gerais, Brasil; 2 Departamento de Bioquímica e Biologia Molecular, Instituto de Ciências Biológicas, Universidade Federal de Goiás (UFG), Goiânia, Goiás, Brasil; University of California, San Diego School of Medicine, United States of America

## Abstract

**Background:**

The Fungal Genome Initiative of the Broad Institute, in partnership with the *Paracoccidioides* research community, has recently sequenced the genome of representative isolates of this human-pathogen dimorphic fungus: Pb18 (S1), Pb03 (PS2) and Pb01. The accomplishment of future high-throughput, genome-wide, functional genomics will rely upon appropriate molecular tools and straightforward techniques to streamline the generation of stable loss-of-function phenotypes. In the past decades, RNAi has emerged as the most robust genetic technique to modulate or to suppress gene expression in diverse eukaryotes, including fungi. These molecular tools and techniques, adapted for RNAi, were up until now unavailable for *P. brasiliensis*.

**Methodology/Principal Findings:**

In this paper, we report *Agrobacterium tumefaciens* mediated transformation of yeast cells for high-throughput applications with which higher transformation frequencies of 150±24 yeast cell transformants per 1×10^6^ viable yeast cells were obtained. Our approach is based on a bifunctional selective marker fusion protein consisted of the *Streptoalloteichus hindustanus* bleomycin-resistance gene (Shble) and the intrinsically fluorescent monomeric protein mCherry which was codon-optimized for heterologous expression in *P. brasiliensis*. We also report successful GP43 gene knock-down through the expression of intron-containing hairpin RNA (ihpRNA) from a Gateway-adapted cassette (cALf) which was purpose-built for gene silencing in a high-throughput manner. Gp43 transcript levels were reduced by 73.1±22.9% with this approach.

**Conclusions/Significance:**

We have a firm conviction that the genetic transformation technique and the molecular tools herein described will have a relevant contribution in future *Paracoccidioides* spp. functional genomics research.

## Author Summary

Diverse eukaryotes, including various fungi, utilize RNA interference (RNAi) pathways to regulate genome-wide gene expression. Since the initial characterization of these pathways and the demonstration of its artificial induction in the filamentous ascomycete *Neurospora crassa*, RNAi has emerged as the most robust reverse-genetic technique to scrutinize the function of genes and has been increasingly adopted in high-throughput functional genomics in search of new insights into fungal pathobiology. Herein, we have developed appropriate molecular tools and straightforward techniques to streamline the generation of stable loss-of-function phenotypes for the human-pathogen *Paracoccidioides brasiliensis*, which is phylogenetically related to *Blastomyces dermatitidis*, *Coccidioides immitis* and *Histoplasma capsulatum*. Likewise these thermo-dimorphic fungi, *P. brasiliensis* infection in immunocompetent or immunocompromised individuals ensue in a life-threatening systemic mycosis known as Paracoccidioidomycosis.

## Introduction

*Paracoccidioides brasiliensis* is a thermo-dimorphic Ascomycota phylogenetically related to *Blastomyces dermatitidis*, *Coccidioides immitis* and *Histoplasma capsulatum*. Likewise these human-pathogen fungi, inhaled conidia from the saprophytic mycelia form of *P. brasiliensis* undergo a most remarkable morphogenetic conversion into a multinucleated yeast form (or spherules in the particular case of *C. immitis*) [1]. This morphogenesis, which is induced by the human host's temperature, is critical for the successful infection of alveolar epithelium. Progression of the primary lung infection in immunocompetent or immunocompromised hosts ensues in a systemic granulomatous mycosis known as Paracoccidioidomycosis (PCM), which is prevalent in Latin America [2]. Suppression of the host's cell-mediated immune response induced by yet undetermined molecular virulence factors is a hallmark of PCM. Since cellular immunity is the most important mode of protection against the yeast form [3], *P. brasiliensis* poses a serious threat to the health and lives of virtually 10 million infected individuals [4].

A distinctive characteristic of an effective anti-fungal therapy is the targeting of those metabolic processes and other virulence attributes that appear to be unique to fungi. Recently, the Fungal Genome Initiative at the Broad Institute (www.broadinstitute.org), in partnership with the *Paracoccidioides* Research Community, has sequenced the genome of this human-pathogen fungus, laying the foundations for future functional genomics research [5]. The accomplishment of functional genomics in a scenario of high-throughput, genome-wide, research relies upon appropriate molecular tools and straightforward techniques to streamline the generation of stable loss-of-function phenotypes. However, these are still unavailable for *P. brasiliensis*, which precludes the wealth of genetic information publicly available for this pathogenic fungus from being explored further and linked to biological function [6]–[9]. In order to circumvent such limitation, we envisaged RNA interference (RNAi) as a feasible and rational approach to scrutinize or to certify the function of target genes by high-throughput means.

RNAi emerged as the most robust reverse-genetic technique for the modulation or suppression of gene expression in many diverse eukaryotes [10], [11]. Since it is double-stranded RNA (dsRNA) that sets the mechanism of this phenomenon in motion, vectors designed for the *in vivo* transcription of a gene fragment arranged as an inverted-repeat separated by an arbitrary spacer, the RNA of which eventually folds back by itself into a hairpin structure (hpRNA), were proved to induce gene silencing effectively but with variable efficiency [12], [13]. The substitution of the arbitrary spacer for a functional intron, which is excised by the spliceosome promoting loopless hpRNA structures, improved considerably the efficiency of RNAi in plant and invertebrate systems [14]–[16]. Perhaps, the utmost advancement in these vectors was the incorporation of a unique and well-characterized lambda phage site-specific recombination system (Gateway Cloning Technology) for the practical and convenient assemblage of ihpRNA templates regardless of restriction endonucleases and ligases [17]. The ease with which these templates are assembled provides vectors such as pHellsgate or pWormgate, originally developed for *Arabidopsis thaliana* and *Caenorhabditis elegans*, respectively, with outstanding advantages over preceding obsolete systems for high-throughput RNAi applications [16], [18].

Herein, our efforts were focused on the development of a purpose-built cassette for high-throughput RNAi in *P. brasiliensis*, which, likewise pHellsgate and pWormgate, incorporates the Gateway Cloning Technology allowing faster downstream analysis. We also report a label system consisting of mCherry [19]. This intrinsically fluorescent monomeric protein, derived from mRFP1 through directed evolution [20], was codon-optimized to increase translational efficiency, thereby improving its performance as a reporter system for transcriptional regulation or as a molecular beacon in protein fusions for the determination of spatial or temporal location patterns in *P. brasiliensis* yeast cells. The cassette was cloned onto the backbone of a binary vector from the pCAMBIA series for *Agrobacterium tumefaciens*-mediated transformation (CAMBIA, Canberra, Australia), which we successfully adapted for efficient high-throughput gene transfer to yeast cells. Furthermore, Zeocin antibiotic resistance conferred by the soil streptomycete *Streptoalloteichus hindustanus* bleomycin-resistance gene (*Shble*) was introduced as a new positive selection marker for the genetic manipulation of *P. brasiliensis* [21], [22].

## Materials and Methods

### Microorganisms, culture media and culture conditions

*P. brasiliensis* yeast cells of the isolate 18 (*Pb18*), the representative of the major phylogenetic group S1 [23], were routinely cultured on solid brain-heart, dextrose, infusion agar (BHDI) or YPD medium (YPD; 1% yeast extract, 2% peptone, 2% dextrose, pH 7.5) at 36°C and were subcultured every period of 10 days.

The wild-type *Pichia pastoris* X-33 strain (Invitrogen, Life Technologies) was routinely cultured in YPD medium at 28°C. X-33 ZeoR transformants were screened and tested for the Mut^+^ phenotype on solid MD and MM media (Invitrogen, Life Technologies), containing 100 µg/mL Zeocin (Invitrogen, Life Technologies), at 28°C.

The “super-virulent” *A. tumefaciens* EHA105 strain, which contains the disarmed Ti plasmid pTiEHA105 derived from pTiBo542, was obtained from CAMBIA (Canberra, Australia) and used as recipient for binary vectors containing cassettes.

*Escherichia coli* TOP10 and DH10B strains (Invitrogen, Life Technologies) were used for the routine propagation of vectors. The *ccd*B Survival T1R strain (Invitrogen, Life Technologies) was used specifically for the propagation of vectors containing the *ccd*B gene. These *E. coli* strains were cultured in LB medium containing the appropriate antibiotic for the selection of transformants at 37°C. Low salt LB medium (Invitrogen, Life Technologies) was used for the selection and culture of *E. coli* with 25 µg/mL of Zeocin.

### Codon-usage optimization and construction of synthetic mCherry ORF

Initially, a reference data file of 22 complete sequenced protein coding genes (CDS) from *Pb18* (Table S1), encompassing 8,286 codons, was compiled to generate a codon usage table that contained the frequency of each codon as the “per thousand ratio” of that codon (www.entelechon.com/resources/online-tools). This codon usage table was then analyzed for the relative frequencies of synonymous codons for each amino acid and a codon frequency data file was generated (Table S2). On the basis of this codon frequency source file, from which rare codons were eliminated by setting a minimal codon usage frequency threshold, a codon-optimized DNA sequence for mCherry (GenBank Accession No AY678264) was designed using “Gene Composer” software [24]. The codon-optimized DNA sequence was edited to eliminate possible restriction enzyme sites incompatible with the overall cloning strategy, direct sequence repeats, cryptic splice sites, endoribonuclease cleavage sites, consensus sequences which lead eventually to premature transcriptional termination and highly stable mRNA secondary structure such as a hairpin or a pseudo-knot. Furthermore, the GC content of the optimized DNA sequence was adjusted in accordance with the mean GC value determined for the reference CDS (Table S1). Subsequently, a set of 6 plus strand (sense) and 6 minus strand (antisense) oligonucleotides, spanning the codon-optimized mCherry sequence, 80-mer in length with an overlap of 20 nucleotides at each end, were synthesized (Table S3) and the desired DNA template was assembled *in vitro* via PCR, in which these overlapping oligos were extended [25]. Briefly, the above-mentioned oligos were mixed to a final concentration of 1.0 µM and diluted 10-fold in a 50 µL PCR mixture containing 2.5 units of a proofreading DNA polymerase, 0.2 mM dNTP, 2.0 mM MgSO_4_ and a standard buffer (Invitrogen, Life Technologies). The reaction was performed for 9 cycles that consisted of denaturing for 30 s at 94°C, annealing for 30 s at 48°C, and extending for 45 s at 68°C, followed by 9 more cycles of 30 s at 94°C, 30 s at 52°C, and 45 s at 68°C. Finally, another 9 cycles of denaturing for 30 s at 94°C, annealing for 30 s at 60°C, and extending for 45 s at 68°C were carried on. The assembled DNA template of mCherry was then amplified for 29 cycles, each consisting of 30 s at 94°C, 30 s at 52°C, and 45 s at 68°C, employing a standard proofreading PCR mixture and a pair of specific primers at 0.4 µM, cloned into pCR2.1-TOPO vector (Invitrogen, Life Technologies) and sequenced.

### Expression of codon-optimized mChery in *P. pastoris*

In accordance with the manufacturer's instruction manual (EasySelect *Pichia* Expression Kit, Invitrogen, Life Technologies), X-33/pPICZ/mCherry Mut^+^ *P. pastoris* recombinant yeast cell colonies were cultured overnight in 10 mL MGY medium at 28°C, in a shaking incubator (250–300 rpm), until log-phase growth (OD600 = 2–6). The recombinant yeast cells were harvested by centrifuging at 3,000× g for 5 min at room temperature and resuspended to an OD_600_ of 1.0 in MMY medium to induce mCherry expression. These yeast cells were further cultured for 24 h at 28°C under adequate aeration. The cultures were sampled at 12 h and 24 h time points, and the functionality of *P. brasiliensis* codon-optimized mCherry was verified in a flow cytometer.

### Vectors and construction of expression cassettes

The pPICZ-A vector (Invitrogen, Life Technologies) was used for the intracellular heterologous expression of codon-optimized mCherry in *P. pastoris* and was also the source for the *S. hindustanus* bleomycin-resistance gene (*Shble*). All the cassette features were amplified using a standard proofreading PCR mixture and then cloned into pCR2.1-TOPO (Invitrogen, Life Technologies) cloning vector. The pCAMBIA-0380 binary vector (CAMBIA; GenBank Acession No AF234290) was used as backbone for *P. brasiliensis* expression cassettes, which were assembled initially on pUC18 cloning vector (GenBank Accession No L08752) for convenience. The *H. capsulatum* calcium binding protein promoter (Prm_CBP1_) was amplified from pCB301-UGFP [26]. The Gateway reading frame cassette (Rfc), containing *att*R1/*att*R2 recombination sites flanking the *ccd*B and the chloramphenicol resistance genes (*att*R1::*ccd*B::CmR::*att*R2), was amplified from pET-DEST42 destination vector (Invitrogen, Life Technologies). The pDONR/Zeo vector (Invitrogen, Life Technologies) was used to generate *att*L-flanked entry clones.

The molecular biology procedures were conducted according to current protocols described elsewhere and all reagents employed were of the highest-grade commercially available. All the amplified cassette features were confirmed by sequencing data, and their correct position and orientation in the cassettes were checked by PCR and restriction enzyme profiling. The oligonucleotide primers used in the overall cloning strategy of these cassette features were listed in Table S4.

**Figure 1:**
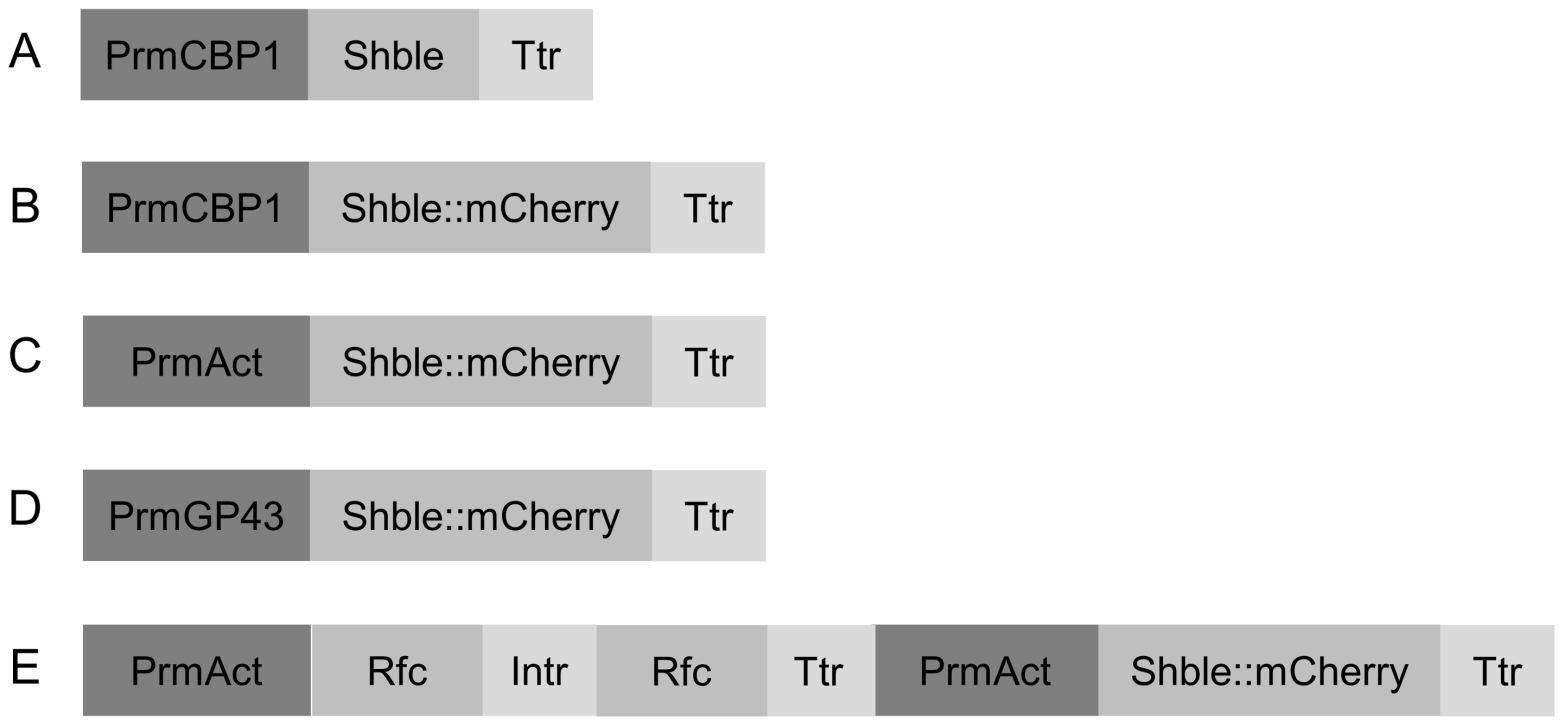
Cassettes assembled in the present work. (**A**). Cassette for the expression of the ZeoR marker under the transcriptional regulation of Prm_CBP1_ and Ttr_GP43_,which was used in preliminary ATMT of *P. brasiliensis*. (**B–D**). Cassettes assembled to validate Prm_Act_ and Prm_GP43_against Prm_CBP1_ through the expression of the bifunctional selective marker “Shble::mCherry” in yeast cells. (**E**). Purpose-built cAlfG, adapted for the Gateway Technology, intended for the expression of intron_GP43_-containing hairpin RNA to trigger RNAi in yeast cells. Abbreviations: Ttr, transcription termination region of *Pb18* GP43 gene; Prm_GP43_, promoter region of *Pb18* GP43 gene; Prm_Act_, promoter region of *Pb18* actin gene; Prm_CBP1_, promoter region of *H. capsulatum* CBP1 gene; Rfc, gateway reading frame cassette; Intr, first intron of the *Pb18* GP43 gene.

The bifunctional selective marker was constructed by fusing the *Shble* ORF to the 5′ extremity end of mCherry ORF. To put it briefly, the downstream region with respect to the 3′ end of the GP43 gene ORF (GenBank Accession No U26160), which contains potential transcription termination and polyadenilation motifs (Ttr_GP43_) [27], was amplified with Ttr_GP43_-F/R primers from *Pb18* genomic DNA and cloned into pCR2.1-TOPO vector. Subsequently, Ttr_GP43_ was transferred to pUC18 cloning vector into *Pst*I/*Hind*III restriction sites, which were previously digested with their respective enzymes. The codon-optimized mCherry ORF was excised with *Sal*I/*Pst*I enzymes from pCR2.1-TOPO vector and cloned into pUC18-Ttr_GP43,_ which had been previously digested with the same pair of restriction enzymes. The *Shble* ORF was then amplified with *Shble*-F/R, excised with *Sal*I/*Xho*I enzymes, and cloned into pUC18-mCh::Ttr_GP43_ at *Sal*I site. Afterwards, Prm_Act_-F/R and Prm_GP43_-F/R primers were used to amplify, respectively, the promoter regions of the Actin gene (Prm_Act_) (GenBank Accession No AY383732) and of the GP43 gene (Prm_GP43_) from *Pb18* genomic DNA. Both promoter regions were cloned into pUC18-*Shble*::mCh::Ttr_GP43_ at *Bam*HI/*Sal*I sites. The promoter of the *H. capsulatum* CBP1 gene (Prm_CBP1_) was amplified with Prm_CBP1_-F/R from pCB301-UGFP vector and, in turn, cloned into pUC18-*Shble*::mCh::Ttr_GP43_. Finally, these assembled cassettes were excised from pUC18 with *Bam*HI/*Hind*III enzymes and cloned into pCAMBIA-0380 (pC0380) at their respective restriction sites (Figure 1A–D).

The ihpRNA expression cassette (Figure 1E) was constructed in accordance with guidelines described elsewhere [18]. Refer to Text S1 for the detailed construction of the ihpRNA cassette.

### BP and LR recombination reactions to construct inverted-repeat GP43 DNA templates for the expression of ihpRNA

*Pb18* yeast cells were cultured until late log-phase growth (∼84 h) in liquid YPD medium, 5% fetal bovine serum (FBS; Gibco, Life Technologies), at 36°C and under adequate aeration in a mechanical shaker. Yeast cells were collected from the upper culture phase (500 µL) and centrifuged. Subsequently, these were suspended in TRIzol Reagent (Invitrogen, Life Technologies) for total RNA extraction in accordance with the manufacturer's instruction manual. The SuperScript First-Strand Synthesis System for RT-PCR (Invitrogen, Life Technologies) was then employed to synthesize the first-strand cDNA from the total RNA. A GP43 DNA segment of 0.5 Kb in relation to the 5′ end of the GP43 mRNA was amplified from the *Pb18* cDNA molecule collection, with the pair of primers GTW.GP43-F/R (Table S4), employing a standard proofreading PCR mixture. This GP43 *att*B1/*att*B2-flanked PCR product was purified and used as *att*B substrate in the BP recombination reaction in accordance with the Gateway manufacturer's instruction manual (Invitrogen, Life Technologies). To put it briefly, an equimolar amount (100 fmol) of GP43 PCR product flanked by *att*B1/*att*B2 recombination sequences and pDONR/Zeo donor vector (Invitrogen, Life Technologies) were added to a standard BP Clonase recombination reaction mixture and incubated at 25°C for 1 h. After this period, proteinase K solution was added to the reaction and further incubated for 10 min at 37°C. Subsequently, 1 µL of this BP recombination reaction was used to transform electrocompetent TOP10 *E. coli* cells. Transformed cells were selected with the appropriate antibiotic (50 µg/mL Zeocin) and screened for entry clones.

For the LR recombination reaction, 100 fmol of pDONR/Zeo-GP43, flanked by *att*L1/*att*L2 recombination sequences, and pC0380-Prom_Act_::Rfc::Intr_GP43_::Rfc::Ttr_GP43_::Prom_Act_::*Shble*::mCh::Ttr_GP43_ purified plasmid DNA were added to standard LR Clonase recombination reaction mixture and incubated at 25°C for 1 h. Proteinase K solution was then added and the reaction was incubated for another 10 min at 37°C. Next, 1 µL of this LR reaction was used to transform electrocompetent TOP10 *E. coli* cells. Transformed cells were selected with the appropriate antibiotic (50 µg/mL kanamycin) and screened for clones. Afterwards, the correct orientation of the GP43 DNA segments in the ihpRNA expression cassette was verified by PCR and restriction enzyme profiling.

### *Agrobacterium tumefaciens*-mediated transformation

*A. tumefaciens* EHA105 transformants, previously selected for the retention of the Ti and binary plasmids, were cultured overnight at 28°C in 5 mL of YEP medium, containing the appropriate antibiotics (20 µg/mL rifampicin; 50 µg/mL kanamycin), under adequate aeration [28], [29]. *A. tumefaciens* cells were then diluted 1∶100 into 5 mL of AB-sucrose minimal medium, pH 5.5, containing the above-mentioned antibiotics, and cultured overnight at 28°C until late log-phase growth, which corresponds to OD_600_ 0.8 (1×10^9^ cells/mL) [28], [29]. After vegetative growth, this overnight culture was centrifuged and the cells were suspended in 10 mL of AB-glucose minimal medium, pH 5.5, containing 200 µM of freshly prepared acetosyringone (Sigma-Aldrich) for *vir* gene induction (AB-induction medium) and antibiotics. This culture was maintained overnight at 25°C under gentle shaking. After induction, the cells were centrifuged, washed with and suspended in 10 mL AB-induction medium [28], [29].

Simultaneously to *A. tumefaciens vir* gene induction, *Pb18* yeast cells were cultured until late log-phase growth (∼84 h) in liquid BHDI or YPD medium, 5% fetal bovine serum (FBS; Gibco, Life Technologies), at 36°C and under adequate aeration in a mechanical shaker. Before these cells were collected from the upper culture phase, debris and larger cell aggregates were allowed to settle. The yeast cells in suspension were collected and centrifuged, washed with and suspended in AB-induction medium. Cell viability was determined by the modified vital dye Janus Green staining method through direct microscopic count in Neubauer counting chamber [30]. Only suspensions of yeast cells with cell viability of ≥90% were used.

The susceptibility of *Pb18* to Zeocin was verified by plating 1×10^6^ viable yeast cells onto BHDI or YPD, 5% FBS, containing this antibiotic at concentrations ranging from 50 to 200 µg/mL. The selective plates were incubated at 36°C until colonies were visible in the negative control and the minimal inhibitory concentration (MIC) was determined as the lowest Zeocin concentration required for total inhibition of yeast cell growth. Thereafter, 1×10^6^ viable yeast cells in AB-induction medium were mixed with induced *A. tumefaciens* cells at ratios of 1∶1, 1∶10 or 1∶100 to a final volume of 2 mL in a 6-well culture plate and this cell mixture was co-cultivated at 25°C for 48 h under gentle shaking. After co-cultivation, 2 mL of BHDI or YPD, 10% FBS, containing 20 µg/mL of gentamicin, was added and this culture was maintained for another 48 h at 36°C under adequate aeration to allow the expression of the *Shble* gene and to inhibit *A. tumefaciens* growth. After this period, the yeast cells were collected, centrifuged, suspended in 200 µL of residual medium and plated onto BHDI or YPD, 5% FBS, and 10 µg/mL of gentamicin, 100 µg/mL of Zeocin. The selective plates were cultured at 36°C until *Pb18* transformants were visible (∼15–25 days). Isolated *Pb18* transformant colonies were then transferred to liquid BHDI or YPD, 5% FBS, and 10 µg/mL of gentamicin, 100 µg/mL of Zeocin, in 12-well culture plate and subcultured every period of 3 days for 3 consecutive rounds to eliminate residual yeast cells containing untransformed nuclei and unstable integrants. Afterwards, these *Pb18* transformant colonies were then transferred to non-selective BHDI or liquid YPD, 5% FBS, in 12-well culture plate and subcultured every period of 3 days for another 3 consecutive rounds to evaluate mitotic stability. The capability for growth on selective liquid BHDI or YPD, 5% FBS, 100 µg/mL of Zeocin, was then evaluated in a 12-well culture plate.

### Direct PCR confirmation of *P. brasiliensis* transformants

The proper insertion of the expression cassettes into the genome of *P. brasiliensis* and its retention were confirmed by a simplified direct PCR protocol. To put it briefly, 10 µL of a ∼84 h yeast cell culture sample were transferred to a 1.5 mL microcentrifuge tube, centrifuged, suspended in 50 µL of a 25 mM β-mercaptoethanol, 5 mM Na_2_EDTA, pH 8.0, solution and incubated for 30 min at room temperature. After this pre-treatment, the yeast cell sample was centrifuged and suspended in 50 µL of a 1 mg/mL *Trichoderma harzianum* enzyme solution (Sigma-Aldrich) and incubated for 1 h at 30°C under gentle shaking. The sample was then freezed at −80°C for 10 min, thawed completely at room temperature, and 5 µL of the cell lysate was diluted 10-fold in a 50 µL PCR mixture containing 1.25 units of *Taq* DNA polymerase, 0.2 mM dNTP, 1.5 mM MgCl_2_, a standard buffer, and the pair of specific primers *Shble*-F/R (Table S4) at 0.4 µM. This reaction was cycled 30 times, each cycle consisting of the following parameters: denaturing for 30 s at 95°C, annealing for 30 s at 52°C, and extending for 30 s at 72°C. Initial and final steps consisted of denaturing for 4 min at 95°C and extending for 3 min at 72°C.

### Flow cytometry data acquisition and analysis

The functional expression of the codon-optimized mCherry ORF by *P. pastoris* yeast cells or the insertion of expression cassettes into genomic DNA of *P. brasiliensis* yeast cells was determined via flow cytometry performed on a basic FACSCan flow cytometer equipped with an argon-ion laser emitting a 488 nm beam at 15 mW (BD Biosciences, Franklin Lakes, NJ, USA). At low flow rate, 50,000 events per sample were acquired and a protocol was defined to measure forward scatter (FSC) and side scatter (SSC) on a linear scale and fluorescence (FL3) on a four-decade logarithmic scale. Data were acquired using “CELLQuest PRO 4.0” (BD Biosciences) and analyzed with “Windows Multiple Document Interface for Flow Cytometry 2.8 (WinMDI 2.8)”.

### Confocal microscopy analysis

*Pb18* yeast cells were cultured until late log-phase growth (∼84 h) in liquid YPD medium, 5% fetal bovine serum (FBS; Gibco, Life Technologies), 100 µg/mL of Zeocin, at 36°C and under adequate aeration in a mechanical shaker.

Confocal microscopy was performed on Zeiss LSM 510 META confocal system. Images were acquired using a Plan-Neofluar 40×/1.3 Oil DIC objective. mCherry was excited at 543 nm and visualized in a red detection channel of 565–682 nm.

### Analysis of *Pb*GP43 transcript levels by real time reversed-transcription PCR (qRT-PCR)

Total RNA from control or from transformed yeast cells was extracted with the TRIzol Reagent (Invitrogen, Life Technologies) and, subsequently, reversed-transcribed with the SuperScript III First-strand Synthesis SuperMix for qRT-PCR in accordance with the manufacturer's instruction manual (Invitrogen, Life Technologies).

qRT-PCR was performed with SYBR Green PCR Master Mix (Applied Biosystems, Life Technologies) in the StepOnePlus Real-time PCR System (Applied Biosystems, Life Technologies). Data were normalized to L34 ribosomal protein transcript reference control. The pair of primers was designed such that one of the primers from each pair spanned an intron, thereby preventing amplification from genomic DNA. The primers used were: *Pbr*_GP43_-S 5′-ATCGATCTCCATGGTGTCCC-3′; *Pbr*_GP43_-A, 5′-CTGGTATGGAGGGTTTGT TGA-3′; *Pbr*_L34_-S 5′-TCAATCTCTCCCGCGAATCC-3′; *Pbr*_L34_-A 5′- AGTTGGCG ATTGTTGTGCGG-3′. For each cDNA sample, qRT-PCR was performed in triplicate and a melting curve analysis was performed to confirm single PCR product. The relative standard curve was generated using a pool of cDNA from each condition which was serially diluted (1∶5–1∶625). Relative expression levels of *Pbr*GP43 were calculated using the standard curve method for relative quantification [31].

### Statistical analysis

The results are expressed as mean ± standard deviation of the mean (SD). Student's *t* test was employed to compare experimental groups and a P value under 0.05 was used to indicate a statistically significant difference.

## Results

### Optimization of mCherry ORF for heterologous expression in *P. brasiliensis*

CDS reference sets were compiled for numerous diverse species by the CUTG database (www.kazusa.or.jp/codon) which made publicly available the data concerning codon usage for these organisms [32]. In the particular case of *P. brasiliensis*, the codon usage table was generated given a CDS reference set composed of 163 ORFs (70.017 codons), 65 (or 40%) of which are ORFs considered more than once. For instance, on closer analysis, we noted that 50 (or 31%) of the entries of this set, which totals up to 20.850 codons, are in fact GP43 ORFs from different isolates, thus affecting the relative frequency calculated for the synonymous codons. Moreover, the compilation process does not discriminate a high-degree from a low-degree expressed gene. Therefore, we opted to generate another synonymous codon usage table based upon our own organized CDS reference set (Table S1) composed mainly of 22 genes encoding glycolytic, heat-shock and ribosomal proteins as noted in sets for eukaryotes displaying translational bias such as *Saccharomyces cerevisiae* [33], [34] or *Pichia pastoris* [35]. The CDS of Formamidase, Y20, Hydrophobin 1 and High-affinity copper transporter proteins, known to be over-expressed, were also included in this set [8].

Synonymous codon usage frequencies for *S. cerevisiae* (column *Sce*, Table S2) and *P. pastoris* (column *Ppa*, Table S2) were calculated based upon reference sets composed, respectively, of 263 and 30 highly expressed CDS. When both codon usage tables are compared, *S. cerevisiae* and *P. pastoris* have 12 (or 79%) similar codon preferences. Compared with *S. cerevisiae*, *Pb18* (column *Pbr*, Table S2) has 5 (or 26%); with *P. pastoris*, *Pb18* has 6 (or 32%) similar codon preferences.

A minimal codon frequency threshold of 35% was established to design an optimized mCherry for *Pb18*, as a means to “harmonize” the codon composition and distribution throughout the length of its CDS. After the *in vitro* assembly of the codon-optimized synthetic mCherry ORF (Figure S1), a *P. pastoris* heterologous expression system was employed to certify it's *in vivo* functionality before attempting final expression in *Pb18*. Although *P. pastoris* and *Pb18* have only 32% of similar codon preferences, this percentage was augmented to 63% by pre-setting the codon frequency threshold at 35%.

**Figure 2:**
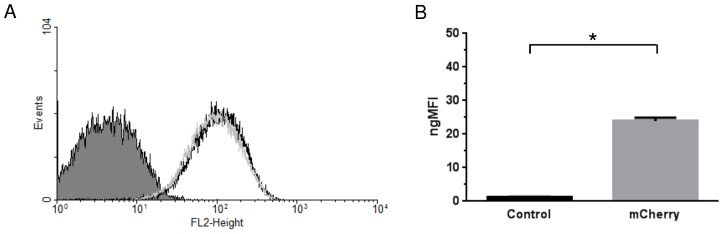
Flow cytometric functional analysis of *P. brasiliensis* codon-optimized mCherry in *P. pastoris*. Synthetic codon-optimized mCherry ORF was expressed in a *P. pastoris* heterologous expression system to certify *in vivo* functionality after the codon-optimization process. (**A**). Representative histogram of the red fluorescence intensity (FL2-Height plotted onto 4 decade logarithmic scale versus frequency of Events) of yeast cell population from two distinct recombinant colonies expressing mCherry (light-gray and black peaks) in respect to a negative control yeast cell population transformed with an empty vector (solid dark-gray peak). (**B**). The geometric mean fluorescence intensity (gMFI) of two positive yeast cell colonies was normalized (ngMFI) in respect to two negative yeast cell colonies and plotted as a bar graph (*, P<0.0001).

Flow cytometric analysis of *P. pastoris* yeast cell populations from two distinct recombinant colonies, positive for the inserted expression cassette, showed a noticeable right shift in red fluorescence intensity (98.89±4.22 gMFI) in comparison to the negative control (4.14±0.52 gMFI) which corresponds to a functional codon-optimized ORF and to the correct translation and folding of mCherry (Figure 2A). The normalized gMFI (ngMFI) was plotted as a bar graph and data analysis confirmed a significant difference (P<0.0001) (Figure 2B).

### High-throughput *A. tumefaciens*-mediated transformation of *P. brasiliensis*

Successful adaptation of *A. tumefaciens*-mediated transformation (ATMT) of *P. brasiliensis* for high-throughput applications required, initially, the careful consideration of a stable resistance marker that would allow the propagation of yeast transformants under a certain selective pressure. We chose to adopt the bleomycin marker encoded by the *ble* gene from *S. hindustanus*, which confers resistance to the broad spectrum antibiotics belonging to the bleomycin/phleomycin family. In our particular case, this marker confers resistance to Zeocin, which binds to and cleaves DNA causing cell death. Since its mode of action differs considerably from that of Hygromycin B, consecutive or even simultaneous antibiotic selection of *Pb18* yeast cells transformed with different cassettes would be possible. Moreover, the *Shble* resistance marker ORF with a length of just 0.375 Kbp would contribute to maintain the overall size of cassettes or binary vectors smaller, thereby facilitating the cloning process.

Susceptibility of *Pb18* to Zeocin was then investigated against antibiotic concentrations ranging from 50 to 200 µg/mL with increments of 50 µg/mL. Complete growth inhibition of 1×10^6^ viable yeast cells was achieved in selective plates containing 100 µg/mL of Zeocin and was thus adopted as the MIC (data not shown).

With the purpose to certify the Zeocin/ZeoR positive selection system for the genetic manipulation of *P. brasiliensis*, a cassette under the transcriptional regulation of the promoter sequence from *H. capsulatum* CBP1 gene (Prm_CBP1_), which was shown recently to be capable of directing transcription in yeast cells [36], and the 3′UTR of the GP43 gene (Ttr_GP43_), which was shown to contain potential transcription termination and polyadenilation motifs [27], was assembled (Prm_CBP1_::*Shble*::Ttr_GP43_) and cloned into pC0380 binary vector (Figure 1A).

**Figure 3:**
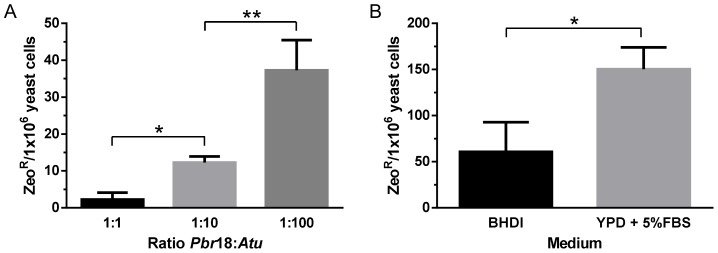
ATMT of *P. brasiliensis* to certify the Zeocin/ZeoR positive selection system and the terminal GP43 gene transcription region. (**A**). Four independent transformation experiments were performed by co-cultivating 1×10^6^ viable *Pb18* yeast cells with *A. tumefaciens* at different ratios. *A. tumefaciens* carried the “Prm_CBP1_::*Shble*::Ttr_GP43_” cassette to express the ZeoR selection marker (*, P = 0.0002; **, P = 0.001). (**B**). Three independent transformation experiments were performed by co-cultivating 1×10^6^ viable *Pb18* yeast cells with *A. tumefaciens* at a ratio of 1∶100 and recovering the yeast cells using two different selective media (*, P = 0.02).

Considering the general consensus on optimal conditions for *vir* gene induction throughout ATMT, late log-phase *Pb18* yeast cells and competent *A. tumefaciens* EHA105, carrying the Prm_CBP1_::*Shble*::Ttr_GP43_ cassette, were co-cultivated at different ratios (1∶1, 1∶10 or 1∶100) for 48 h at 25°C. After the co-cultivation period, BHDI medium was added and yeast cells were cultivated for another 48 h, but at 37°C, to allow their recovery and, more importantly, the expression of the *Shble* gene. Four independent ATMT experiments were performed and data analysis revealed that the highest transformation frequency, 37±8 yeast transformants/1×10^6^ viable yeast cells, was achieved with a ratio of 1∶100 *Pb18*:*At* (Figure 3A). The genomic DNA obtained from transformants was positive for the insertion of the expression cassette in the direct PCR analysis which was confirmed by amplifying the *Shble* segment (data not shown). Our preliminary results showed that Ttr_GP43_, a genomic DNA segment of only 0.325 Kbp in length, was sufficient to terminate transcription and to signal proper polyadenilation of the *Shble* transcript. These results showed also that the positive selection system composed of Zeocin and the ZeoR marker could be employed for gene manipulation, since the level of *Shble* protein produced in yeast cells was apparently sufficient to detoxify Zeocin preventing cell death (Figures S4-A-E) and natural resistant colonies were not observed in control selective plates throughout the experiments (Figure S4-F). Furthermore, controls from these experiments, in which the recovery step was suppressed, led us to conclude surprisingly that the fastidious *Pb18* yeast cells tolerates the conditions imposed by the co-cultivation step of ATMT, more specifically, a poor nutritional and acid (pH 5.5) medium, although cell viability was greatly reduced to 30±9% viable yeast cells (data not shown).

Although 37±8 ZeoR transformants per 1.0×10^6^ viable recipient cells represented an improvement over previously reported results [36], [37], we reasoned that other aspects of the ATMT procedure could be implicated in such low transformation efficiency. Therefore, we decided to further investigate the influence of yeast cell viability, which in fact was remarkably low after the co-cultivation step, on the transformation efficiency.

The plating efficiency of *P. brasiliensis* has been shown to increase considerably through the supplementation of standard mycological medium with horse serum and the supernatant or extract of yeast cultures [38], [39]. By this point, we substituted the BHDI medium used throughout the ATMT procedure for YPD supplemented with 5% of FBS and investigated the effect of recipient yeast cell viability on transformation efficiency (Figure 3B). Indeed, cell viability after the co-cultivation step increased from 50% in BHDI up to 87% in YPD, 5% FBS (data not shown). Just as we conjectured, augmentation of yeast cell viability reflected immediately over the ATMT efficiency and resulted, remarkably, in a transformation frequency of 150±24 ZeoR yeast cells, corresponding to approximately twice the transformation frequency observed for experiments in which BHDI medium was employed (Figure 3B). Altogether, our results suggest that the efficiency of *P. brasiliensis* transformation is also governed by recipient cell viability besides the optimal conditions for *vir* regulon gene induction in *A. tumefaciens*.

### Validation of Actin and GP43 gene promoter regions for heterologous protein expression in *P. brasiliensis*

Heterologous protein expression in *P. brasiliensis* was achieved recently with different promoter regions from *Aspergillus sp*, *H. capsulatum* or *Neurospora crassa* [36], [37], [26]. Since these regions are elements of complex regulatory networks, thereby subjected to differential transcription regulation patterns, we opted for promoters native to *Pb18* as functional regulatory elements of cassettes intended for heterologous protein expression in yeast cells.

The upstream sequences relative to the translation start site of the Actin and the GP43 ORFs of this dimorphic fungus were amplified [37], [40]. The former amplified DNA segment of 0.895 Kbp in length was shorter in comparison to the same genomic DNA region previously reported for strain IVIC *Pb*73. The alignment of these sequences revealed an identity of 97% and a noteworthy deletion of 0.274 Kbp extending from −1169 to −895 (Figure S2). A potential TATA box had been identified within the corresponding genomic DNA segment of *Pb*73 (−961) [40]. Since accurate transcription from various protein-coding genes relies on a TATA box element and given that less than 2% of *S. cerevisiae* genes contains two or more non-overlapping TATA sequences [41], we ascertained the existence of additional potential TATA box elements and other functional DNA motifs referred to as “core promoter elements” in the amplified DNA segment that justified the attempt of functional tests [42], [43]. This DNA segment was analyzed considering that approximately 20% of *S. cerevisiae* genes contains a TATA box, which the consensus sequence is “TATAWAWR”, within a confined upstream location that extends from position −200 to −50 relative to the translation start site [41]. On closer analysis, no other TATA box was identified in accordance with this criterion but a conspicuous TATA-like element, which satisfy the minimum consensus sequence “TAWWWAWR”, was located at −338 (“TAATAATA”) (Figure S2). Besides this sequence, an element positioned at −422 (“TATTTAAG”) also satisfy this minimum consensus. In addition, a non-consensus variant at the sixth and eighth position was localized at −358 (“TAATATAT”) and another variant at the sixth and seventh position at −125 (“TAATATCA”). Notably, the former non-consensus variant was shown to be a functional TATA box element in *S. cerevisiae* actin promoter. Indeed, several studies revealed that a wide variety of A/T-rich sequences can function as TATA boxes [42], [43].

We next surveyed the amplified upstream genomic DNA segment from the Actin ORF for consensus sequences of three related yeast transcription initiator elements (Inr) RRYRR, TCRA and YAWR, where the transcription start site (TSS) is underlined; R is purine and Y is pyrimidine [44]. Five purine (R)-rich elements (RRYRR) were located downstream of a TATA box element at positions −413 (“GGTGG”), −388 (“GGCGG”), −236 (“AACAA”), −205 (“AACAA”) and −118 (“AACAG”). Interestingly, three of these elements are overlapped by a TCRA element of sequence “TCAA” (−238, −207 and −120 respectively); an additional non-overlapping element was located at −214 (“TCAA”). Moreover, three sequences consistent with YAWR were located at −363 (“TATA”), −286 (“CAAA”) and at −258 (“CAAG”) (Figure S2). The TATA to TSS distance in *S. cerevisiae* has been reported to range from 45 to 120 nt, thus with the exception of the transcription initiator elements positioned at −413, −120 and −118, all other elements fall within this distance downstream from a TATA box (Figure S2) [45]. Furthermore, a long R.Y (purine.pyrimidine) tract downstream of the abovementioned core promoter elements was located extending from −97 to −59, consistent with previous observations that such binary DNA tract is enriched in promoter regions of eukaryotes and might function as DNA unwinding elements (DUE) [46].

This same analysis was performed for the amplified upstream DNA segment of 0.320 Kbp relative to the translation start site of GP43 ORF (Figure S3). Our observations confirm and complement previous identification of potential core promoter elements in the corresponding genomic DNA of *Pb*B339 [27]. A canonical TATA box element was also located at −80 (“TATAAATA”) and precedes an immediate R.Y tract that extends from −65 to −39. The YAWR positioned at −25 (“CAAG”) was also identified besides another element at −10 (“CATA”). Moreover, an overlapping TCRA element was located at −26 (“TCAA”) (Figure S3).

Since these genomic DNA segments amplified from the promoter regions of the Actin and GP43 genes contained potential core promoter elements, which direct the recruitment and assembly of the class II basal transcription factors, including RNAP II, into a functional pre-initiation complex [42], we investigated whether or not the transformation efficiency of ATMT could be improved further by regulating the transcription of the ZeoR selection marker with Prm_Act_ or Prm_GP43_. For this investigation, the codon-optimized ORF of mCherry was fused to the 3′ extremity end of ZeoR with the purpose of creating a bifunctional selective marker that would couple antibiotic and fluorescence screening of transformants (Figure 1B–D).

**Figure 4:**
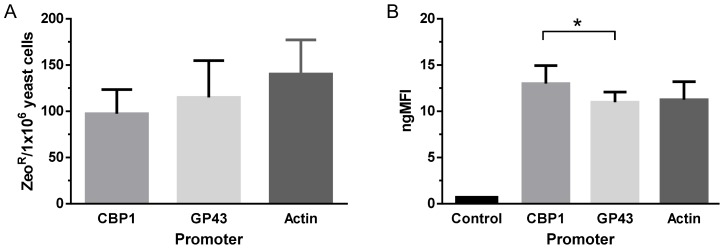
*A. tumefaciens* mediated transformation efficiency of *Pb18* yeast cells with cassettes under the transcriptional regulation of different promoters. (**A**). Three independent ATMT experiments were performed to verify if the transformation efficiency of *Pb18* yeast cells could be further improved by expressing the bifunctional selective marker under the transcriptional regulation of Prm_Act_ or Prm_GP43_ in relation to Prm_CBP1_. Data analysis showed non-significant differences (CBP1 vs. Actin, P = 0.17; CBP1 vs. GP43, P = 0.55). (**B**). The gMFI of the bifunctional selective marker “Shble::mCherry”, which was expressed under the transcriptional regulation of Prm_CBP1_, Prm_Act_ or Prm_GP43_, was determined for yeast cell populations in late log-phase growth. Control refers to yeast cell populations transformed with the cassette “Prm_CBP1_::*Shble*::Ttr_GP43_”. The gMFI values were normalized in respect to control yeast cell populations expressing only the ZeoR selection marker. (*, P = 0.01).

Although an apparent higher transformation frequency was observed for yeast cells transformed with the cassette under the regulation of Prm_Act_ or Prm_GP43_ in comparison to the cassette regulated by Prm_CBP1_, statistical analysis showed non-significant differences (Figure 4A). The mitotic stability of 10 *Pb18* yeast cell colonies transformed with the Prm_Act_::*Shble*::mCh::Ttr_GP43_ cassette was evaluated and, as a result, 80% (or 8 yeast cell colonies) were stable. Interestingly, when 10 stable yeast clones from *Pb18* populations transformed with Prm_CBP1_::*Shble*::mCh::Ttr_GP43_ were subjected to a higher concentration of antibiotic from 100 µg/mL up to 200 µg/mL, only 30% (or 3 yeast transformants) of them were able to detoxify Zeocin and survive. The contrary was noted for the stable yeast clones transformed with Prm_Act_::*Shble*::mCh::Ttr_GP43_ or Prm_GP43_::*Shble*::mCh::Ttr_GP43_ of which 70% (or 7) and 50% (or 5) respectively survived the higher antibiotic concentration (data not shown). These results suggested distinct levels of bifunctional selective marker expression during yeast cell cycle due possibly to intrinsic differential transcription regulation of promoter regions or due to other factors arising from the penetrance variability of these cassettes. To verify this possibility, we analyzed the mCherry gMFI of these stable *Pb18* clone populations transformed with each cassette in question at late log-phase growth by flow cytometry. No significant difference was revealed through statistical analysis (P = 0.69) comparing the gMFI of mCherry expressed under the transcriptional regulation of Prm_CBP1_ (270.4±40.7 gMFI) to Prm_Act_ (234.3±40.4 gMFI). When compared to Prm_GP43_ (223.10±23.6 gMFI), mCherry gMFI from Prm_CBP1_ was significantly higher (P = 0.01) (data not shown). These gMFI results were normalized in respect to control yeast cell populations expressing only the ZeoR selection marker (Prm_CBP1_::*Shble*::Ttr_GP43_; Figure 1A). Under the transcriptional regulation of Prm_Act_ and Prm_GP43_, yeast cell populations were 11.3±1.9 and 11.0±1.1 times more fluorescent than the control yeast cell populations; Prm_CBP1_ yeast cell populations were 13.0±2.0 times more fluorescent (Figure 4B).

**Figure 5:**
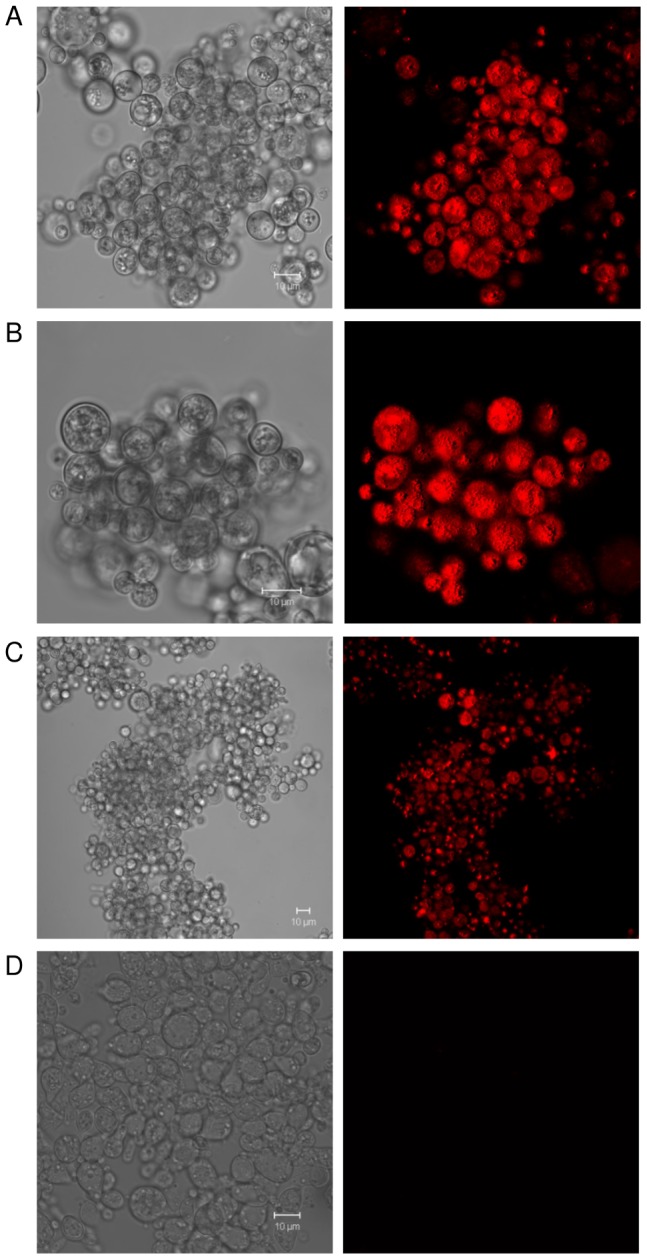
Epifluorescence microscopy analysis of yeast cells expressing the bifunctional selective marker. Yeast cells carrying integrated cassettes under the transcriptional regulation of Prm_CBP1_ (**A**), Prm_Act_ (**B**) or Prm_GP43_ (**C**) were collected from late-log phase cultures and visualized through confocal microscopy. (D). Yeast cells carrying the integrated “Prm_CBP1_::*Shble*::Ttr_GP43_” expression cassette was used as negative control. Scale bar: 10 µm.

The cell compartmentalization of the bifunctional selective marker, visualized by confocal microscopy, revealed a rough brilliant red fluorescence evenly distributed throughout the cytoplasm of both mother and daughter yeast cells in all transformants analyzed (Figure 5A–C). Interestingly, higher fluorescence intensities were frequently co-localized with vacuolar-like structures due possibly to sequestered *Shble*::mCherry fusion protein.

### Induction of RNAi in *Pb18* yeast cells through the expression of ihpRNA aimed to knock-down the GP43 gene

**Figure 6:**
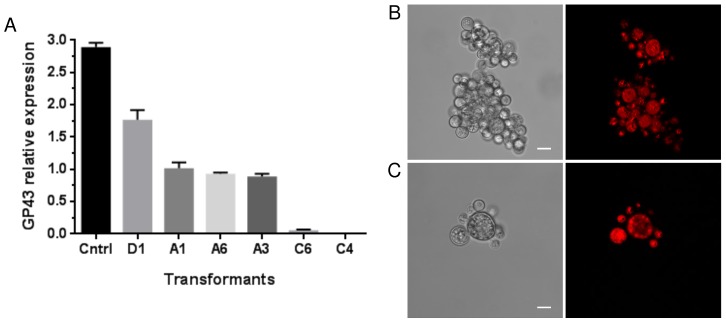
RNA interference of *Pb18* GP43 gene expression triggered artificially with intron-containing dsRNA. (**A**). GP43 gene expression levels were investigated in six cAlf-GP43 transformants in relation to cAlfG-control by qRT-PCR after 8 months of subculturing in non-selective media. Results were normalized against the internal control L34. (**B–C**) Epifluorescence microscopy analysis of transformant C4 (C), in which the GP43 gene expression was nearly abolished, in comparison to cAlfG-control (**B**). Scale bar: 10 µm.

An ihpRNA expression cassette (cAlfG), which incorporates the Gateway Cloning Technology to streamline the assembly of inverted-repeat DNA templates spaced by an intron derived from the GP43 gene, was assembled (Figure 1E). In order to knock-down GP43 gene expression in wild type *Pb18* (WT-*Pb18*), yeast cells were transformed, in accordance with the ATMT procedure established in the present work, with cAlfG-GP43 to express an ihpRNA with a length of 0.5 Kb. For control, WT-*Pb18* yeast cells were transformed with an empty cAlfG. Proper cAlfG-GP43 insertion into genomic DNA and stability were verified and confirmed indirectly by screening transformants for mCherry expression by fluorescence microscopy. Six red fluorescent transformants were randomly selected for further analysis to investigate if relative GP43 gene expression levels were knocked-down by RNAi and to what extent (Figure 6A). Indeed, qRT-PCR analysis, performed eight months after ATMT, showed that GP43 transcript levels in these transformants were reduced to a mean 26.9±22.9% in comparison to empty cAlfG control. Noteworthy, we were able to select two transformants, C6 and C4, in which the expression of the GP43 gene was nearly abolished (Figure 6A). In these particular transformants, gp43 transcript levels were reduced respectively by 98.0±0.4% and 99.9±0.01%. Visualization of the C4 transformant through confocal microscopy didn't reveal any apparent morphological alteration due to GP43 gene knock-down (Figure 6C).

## Discussion

In the past decade, the transcriptome profiling undertaken by the *Paracoccidioides* Transcriptome Consortium was a landmark in the ongoing quest for genes differentially expressed in the mycelium and yeast forms, genes relevant for host-pathogen interactions, genes related to potential drug targets in other model dimorphic fungi and novel genes for which a biological function is yet unknown [6]–[9], [47], [48]. Recently, the Fungal Genome Initiative of the Broad Institute, in partnership with the *Paracoccidioides* Research Community, has sequenced the genome of this pathogenic fungus [5]. Paradoxically, development of the molecular tools or the technical procedures indispensable to scrutinize the function of each of these and the remaining unknown genes by high-throughput means to reveal new insights into fungal pathobiology hasn't occurred concomitantly.

The versatile *A. tumefaciens* mediated transformation system, which was originally developed for plant species and has since been successfully adapted to other diverse species, including fungi [49]–[51], was shown to alter *P. brasiliensis* genetically, albeit with a low transformation frequency (3 HygR/10^6^ yeast cells) [37]. Almeida *et al* [36] investigated several critical parameters that are known to affect the efficiency of this system and showed, most importantly, that insertion of a GFP expression cassette into *P. brasiliensis* haploid genome occurred invariably once. Although the transformation frequency was slightly ameliorated by these authors (5±1 HygR/10^6^ yeast cells), it still precluded any immediate functional genomics study on a grand scale similarly to the one conducted recently for the rice blast fungus, *Magnaporthe oryzae*, in which>20,000 mutants were generated by insertional mutagenesis revealing the genetic basis of its pathogenicity [52], [53].

In the present work, we not only have achieved a higher transformation frequency, we have also shown that induced *A. tumefaciens* attaches to and transfers T-DNA to *Pb18* yeast cells successfully when co-cultivated in suspension. The routine procedure is to carry out co-cultivation of induced *A. tumefaciens* and recipient cells on nitrocellulose membrane placed onto solid induction medium [50] which is per se awkward when handling fungal species that poses a threat to biosafety, *P. brasiliensis* as such. Furthermore, co-cultivation performed in liquid induction medium confers ease and flexibility to this system since, for example, multiple ATMT reactions could be performed simultaneously in multi-well culture plates in the case of high-throughput RNAi or a co-culture could be easily scaled according to a desired number of transformants in the case of the construction of an insertional mutant library. In preliminary transformations of *P. brasiliensis*, we focused on the optimal conditions for maximum and consistent *vir* regulon gene induction by acetosyrongone, which have been investigated exhaustively elsewhere and reviewed recently [29]. In accordance to the general consensus, maximum induction occurs in an acid (∼pH 5.2–6.0) defined medium containing a “co-inducer” sugar, glucose, at 25°C. By the end of the induction step, *A. tumefaciens* has processed the T-DNA on the binary plasmid, assembled the type IV secretion system and is competent to transfer T-DNA and Vir proteins as soon as it attaches to a recipient cell. Since the subpopulation of homokaryon yeast cells is significantly higher in comparison to the subpopulation of heterokaryon yeast cells in late log-phase (72–84 h) batch cultures [54], *Pb18* yeast cells cultured until late log-phase growth were chosen as recipients. We reasoned that the selection of homokaryon transformants after ATMT would be more effective by adopting this strategy despite the fact that multinucleate budding mother cells generate daughter cells with one nucleus. Interestingly, in the case of the multinucleate dimorphic fungus *B. dermatitidis*, the first round of selection after co-cultivation results in a transformant population characterized by homokaryon yeast cells [51]. Our initial approach to ATMT of *P. brasiliensis* resulted in a fairly higher transformation frequency of 37±4 ZeoR/10^6^ yeast cells, in comparison to abovementioned previous reports, but still would hinder a more ambitious functional genomics study. Low yeast cell viability was particularly noticeable after the co-cultivation step throughout preliminary ATMT, what led us to conjecture that substantial numbers of competent *A. tumefaciens* bacterial cells could have attached and attempted T-DNA transfer to nonviable yeasts, thereby reducing the frequency of effective transformation. Therefore, we investigated the impact of yeast cell viability on transformation frequency by raising it considerably through the substitution of BHDI growth and selective medium for a richer complex medium (YPD) supplemented with 5% fetal bovine serum (FBS). Indeed, Castaneda *et al* [38] have shown that a standard mycological medium such as BHDI is ordinarily inferior in comparison to the modified McVeigh/Morton medium and that its supplementation with 4% horse serum combined with 5% culture filtrate from stationary phase yeast cells markedly improved *P. brasiliensis* plating efficiency. Moreover, Kurita *et al* [39] also improved the plating efficiency of *P. brasiliensis* up to 94–99% by supplementing BHDI with 4% horse serum in combination with 5% of a yeast cell water-extract. Remarkably, substitution of the BHDI medium used throughout the ATMT procedure for YPD, supplemented only with 5% FBS, reflected instantly on the augmentation of transformation frequency: 150±24 ZeoR yeast cells. Afterwards, it would be extremely relevant to verify the effect of synergy between FBS and yeast cell extract or culture filtrate on the transformation frequency. Indeed, factors in the yeast cell extract or culture filtrate, such as growth-enhancement factors, might assist to re-enact the yeast form genetic program faster once *P. brasiliensis* is switched to the recovery stage of ATMT at 36°C, after co-cultivation which is performed at 25°C for 48 h.

RNAi has become the reverse-genetic technique of choice to reliably knock-down genes in many diverse eukaryotes. It has also proven to be an effective technique when applied in scenarios of high-throughput, functional-genomic, studies [55], [56]. Since the initial characterization of RNAi pathways and the demonstration of its artificial induction in the filamentous ascomycete *N. crassa* [57], RNAi has increasingly been adopted for fungi [58]–[64]. Typically, in these microorganisms, RNAi is artificially induced via host cell's transcriptional machinery which produces a RNA precursor homologous to a target gene from an integrative or auto-replicative DNA element. This precursor eventually folds into a hairpin RNA structure (hpRNA) or forms dsRNA by hybridizing to sense-transcripts from the target gene. This later approach, designated antisense RNA technology (aRNA), has been implemented recently to knock-down CDC42 [65], HAD32 [66], AOX [67], HSP90 [68], GP43 [69] and Rtb5 [70] genes in *Paracoccidioides* spp., although the most potent RNAi trigger in fungi are promoter expressed hpRNAs extending from 0,2 to 0,5 Kb in length [71]. The rationale underlying the efficiency with which intron-containing hairpin RNA (ihpRNA) triggers RNAi is that the spliceosome promotes perfect alignment and then hybridization of inverted-repeat sequences during the intron excision process whereas the hybridization of an antisense RNA to its sense counterpart occurs entirely by chance. This alternative approach has improved considerably the extent of target gene knock-down in invertebrate, plant and filamentous fungus model organisms. Hence, we have developed a purpose-built cassette for high-throughput RNAi in *Paracoccidioides* ssp., which, likewise pHellsgate, pWormgate and, recently, pTroya [72], incorporates the Gateway Cloning Technology to streamline the construction of intron-containing, inverted-repeat, DNA templates. Indeed, with cAlfG-GP43, we were able to obtain *Pb18* stable transformants in which the GP43 gene expression was nearly abolished. Moreover, since its expression level was reduced to various extents, transformants presenting a spectrum of phenotypes were a possibility, although herein not further verified. Yeast cells could have presented strong variability due to the dynamic adaptations constantly occurring in response to its ever-changing microenvironment, which is determined by the cell population context. This context influences the expression of a large number of genes, metabolic activity and signal transduction, which could have interfered in the knock-down efficiency of the GP43 gene expression in certain transformant yeast cell populations [73].

In fungi, knock-down of gene expression by RNAi is inherently variable. Coupling the knock-down of a target gene with that of a marker gene, as a means to report the extension of RNAi, could assist in the selection of highy silenced transformants. To overcome the challenge of such variability, vector systems in which the expression of an intrinsically fluorescent protein gene, like mCherry for instance, is concomitantly silenced with a target gene, employing a chimeric hpRNA, have been developed. Knocked-down transformants presenting the non-fluorescent phenotype can then be screened with ease through fluorescence microscopy [59].

## Supporting Information

Figure S1 **Alignment of the original (Ori) and the optimized (Opt) coding DNA sequence versions of mCherry.** Codons in mCherry's CDS that were adjusted in accordance with *Pb18* codon usage preferences are shaded in dark gray.
(DOCX)

Figure S2 **Alignment of** ***Pb18*** **Actin gene promoter region to the corresponding genomic DNA of IVIC** ***Pb*** **73.** Light shading denotes identical nucleotides. Identified potential core promoter elements are marked as follow: TATA box elements are dark shaded and transcription initiator elements are typed in bold. The R.Y tract element is typed in italic. The corresponding deleted sequence is underlined while Prm_Act_-F/R annealing sequences are cross-lined.
(DOCX)

Figure S3 **Alignment of** ***Pb18*** **GP43 gene promoter region to the corresponding genomic DNA of IVIC** ***Pb*** **73.** Light shading denotes identical nucleotides. Identified potential core promoter elements are marked as follow: TATA box elements are dark shaded and transcription initiator elements are typed in bold. The R.Y tract element is typed in italic.
(DOCX)

Figure S4 **Validation of the Zeocin/ZeoR positive selection system for the genetic manipulation of** ***P. brasiliensis*** **.** *Pb18* yeast cell colonies (white arrows) expressing the ZeoR selection marker from the “Prm_CBP1_::*Shble*::Ttr_GP43_” cassette inserted into genomic DNA by ATMT. (**A–E**). Light microscope images of viable and stable *Pb18* transformants growing on the surface of selective medium at 72 h (3 days), 120 h (5 days) and 168 h (7 days) after the recovery cultivation step; amplified 40×. (**F**). Representative image of control yeast cells at 168 h (7 days), transformed with an empty pCAMBIA0380 vector, at 168 h of growth in selective medium; amplified 40×.
(DOCX)

Table S1 **Reference set of CDS used to determine codon usage preferences for** ***P. brasiliensis*** **.**
(DOCX)

Table S2 **Comparison of synonymous codon frequency in highly expressed CDS.**
(DOCX)

Table S3 **Set of oligonucleotides for the** ***in vitro*** **assembly of synthetic codon-optimized mCherry DNA template.**
(DOCX)

Table S4 **Set of oligonucleotides for the construction of expression cassettes.**
(DOCX)

Text S1 **Expression cassette assembly.**
(DOCX)
