# Supplementary material for: New Developments of RNAi in Paracoccidioides brasiliensis: Prospects for High-Throughput, Genome-Wide, Functional Genomics
Source: PLoS Negl Trop Dis. 2014 Oct 2;8(10):e3173. doi: 10.1371/journal.pntd.0003173 (PMC4183473; doi:10.1371/journal.pntd.0003173)
Supplement: Figure S1 — Alignment of the original (Ori) and the optimized (Opt) coding DNA sequence versions of mCherry. Codons in mCherry's CDS that were adjusted in accordance with Pb18 codon usage preferences are shaded in dark gray. (DOCX) [file pntd.0003173.s001.docx]

Supporting information: Figure S1.

1 M V S K G E E D N M A I I K E F M R F K V H M E G S V N G H

Ori 1 ATG GTG AGC AAG GGC GAG GAG GAT AAC ATG GCC ATC ATC AAG GAG TTC ATG CGC TTC AAG GTG CAC ATG GAG GGC TCC GTG AAC GGC CAC

Opt ATG GTC TCC AAG GGT GAA GAA GAT AAC ATG GCT ATC ATC AAG GAA TTC ATG CGT TTC AAG GTC CAC ATG GAA GGT TCC GTC AAC GGT CAT

31 E F E I E G E G E G R P Y E G T Q T A K L K V T K G G P L P

91 GAG TTC GAG ATC GAG GGC GAG GGC GAG GGC CGC CCC TAC GAG GGC ACC CAG ACC GCC AAG CTG AAG GTG ACC AAG GGT GGC CCC CTG CCC

GAG TTC GAG ATC GAA GGT GAA GGT GAA GGT CGT CCT TAC GAG GGT ACC CAA ACC GCT AAG CTC AAG GTC ACC AAG GGT GGT CCT CTC CCT

61 F A W D I L S P Q F M Y G S K A Y V K H P A D I P D Y L K L

181 TTC GCC TGG GAC ATC CTG TCC CCT CAG TTC ATG TAC GGC TCC AAG GCC TAC GTG AAG CAC CCC GCC GAC ATC CCC GAC TAC TTG AAG CTG

TTC GCT TGG GAT ATC CTC TCC CCT CAA TTC ATG TAT GGT TCC AAG GCT TAC CGT AAG CAC CCT GCT GAT ATC CCT GAT TAC CTC AAG CTC

91 S F P E G F K W E R V M N F E D G G V V T V T Q D S S L Q D

271 TCC TTC CCC GAG GGC TTC AAG TGG GAG CGC GTG ATG AAC TTC GAG GAC GGC GGC GTG GTG ACC GTG ACC CAG GAC TCC TCC CTG CAG GAC

TCC TTC CCT GAA GGT TTC AAG TGG GAA CGT GTC ATG AAC TTC GAA GAT GGT GGT GTC GTC ACC GTC ACC CAG GAC TCC TCC CTC CAA GAC

121 G E F I Y K V K L R G T N F P S D G P V M Q K K T M G W E A

361 GGC GAG TTC ATC TAC AAG GTG AAG CTG CGC GGC ACC AAC TTC CCC TCC GAC GGC CCC GTA ATG CAG AAG AAG ACC ATG GGC TGG GAG GCC

GGT GAA TTC ATC TAT AAG GTC AAG CTC CGT GGT ACC AAC TTC CCT TCC GAC GGC CCC GTA ATG CAG AAG AAG ACC ATG GGT TGG GAG GCT

151 S S E R M Y P E D G A L K G E I K Q R L K L K D G G H Y D A

TCC TCC GAG CGG ATG TAC CCC GAG GAC GGC GCC CTG AAG GGC GAG ATC AAG CAG AGG CTG AAG CTG AAG GAC GGC GGC CAC TAC GAC GCT

TCC TCC GAA CGT ATG TAC CCT GAG GAC GGT GCT CTC AAG GGT GAG ATC AAG CAG CGT CTC AAG CTC AAG GAC GGT GGT CAC TAC GAC GCT

181 E V K T T Y K A K K P V Q L P G A Y N V N I K L D I T S H N

451 GAG GTC AAG ACC ACC TAC AAG GCC AAG AAG CCC GTG CAG CTG CCC GGC GCC TAC AAC GTC AAC ATC AAG TTG GAC ATC ACC TCC CAC AAC

GAG GTC AAG ACC ACC TAT AAG GCT AAG AAG CCT GTC CAG CTC CCT GGT GCT TAT AAT GTC AAT ATC AAG CTC GAT ATC ACC TCC CAT AAT

211 E D Y T I V E Q Y E R A E G R H S T G G M D E L Y K *

541 GAG GAC TAC ACC ATC GTG GAA CAG TAC GAA CGC GCC GAG GGC CGC CAC TCC ACC GGC GGC ATG GAC GAG CTG TAC AAG TAA

GAG GAT TAC ACC ATC GTC GAG CAG TAC GAG CGT GCT GAG GGT CGT CAT TCC ACC GGT GGT ATG GAT GAG CTC TAT AAG TAG

**Figure S1. Alignment of the original (Ori) and the optimized (Opt) coding DNA sequence versions of mCherry**. Codons in mCherry’s CDS that were adjusted in accordance with *Pb18* codon usage preferences are shaded in dark gray.
