# Supplementary material for: New Developments of RNAi in Paracoccidioides brasiliensis: Prospects for High-Throughput, Genome-Wide, Functional Genomics
Source: PLoS Negl Trop Dis. 2014 Oct 2;8(10):e3173. doi: 10.1371/journal.pntd.0003173 (PMC4183473; doi:10.1371/journal.pntd.0003173)
Supplement: Figure S2 — Alignment of Pb18 Actin gene promoter region to the corresponding genomic DNA of IVIC Pb 73. Light shading denotes identical nucleotides. Identified potential core promoter elements are marked as follow: TATA box elements are dark shaded and transcription initiator elements are typed in bold. The R.Y tract element is typed in italic. The corresponding deleted sequence is underlined while PrmAct-F/R annealing sequences are cross-lined. (DOCX) [file pntd.0003173.s002.docx]

Supporting information: Figure S2.

-100 -80 -60 -40 -20 -1

|.........|.........|.........|.........|.........|.........|.........|.........|.........|........|

*Pb*73 -1200 ~~tcgcccttactatagggcacg~~cgtggtggccctagcttttcatcctttcgaaaaggctgtggagggagaatagattcctcggagcggagaa

*Pb18* -------------------------------------------------------------------------------------------

-1100 gggatgtaacggaggagtagagttatccacattccacaatcctcgcttttctagctgaaataactactgtgcatacaagattgtgtgaatgttagaaagt

----------------------------------------------------------------------------------------------------

-1000 GACCCctgaggccctcaaaggatgtcaacttattgccctttattaaccttcttggaccagtgagctcttttcttgctccctctaccacatacaacgcgcg

----------------------------------------------------------------------------------------------------

-900 TCCC~~CCCGTTAACTACGGGCACG~~AAAAGTGTTAAAAGGTTTGGCGGTTGCCTTGTAGATTCGCACCAGAAGATTCCAGAACGGGAGGTGTTGATGGATCG

----CCCCTTACTATAGGGCACGGAAAGTGTTAAAAGGTTTGGTGGTTGCCTTGTAGATTCGCACCAGAAGATTCCAAAGCGGGAGGTGTTGATGGATCG

-800 GACAATAGGCAGATATCAGTGTTTCATGGGCGAGAATGAAAAGAGAGGTACCAAGAATCTTTTTGCCTTGGATTGGGGACATATTCAGGTTGACTTAGGG

GACAATAGGCAGATATCAGTGTTTCATGGGTGAGAATGAAAAGAGAGGTACCAAGAATCTATTTGCCTTGGATTGGGGATATATTCAGGTTGACTTAGGG

-700 AGTACCACCACGCCGGGCTGTCGTCGTATGGGGACAGACGTACCAAGACGTTTCACTTTTGACCCCGCCATTATGAAAGACACAACATGGCAGATACGGT

AGTACCACCACGCCGGGCTGTCGTTGTATGGGGACAGACGTACCAAGAAGTTTCACTTTTGACCCCGCCATTATGAAAGACGCAACATGGCAGATACGGT

-600 ACACGTATGGTACGATACATCCATCCATTTAGGAGATATGAACGGGTATATGGCATGGAGTAGAGGAATAGCTGAAGAGATGCTTACACCCTGCGTGCCG

ACACGTATGGTACGATTCATCCATCCATTTAGGAGATATGAACGGGTATATGGCATGGAGTAGAGGAATAGCTGAAGAGATGCTTACACCCTGTGTGCCG

-500 CAGAGAATTCGTCATGGTCGTCTTGCCGTCTCTTGTTCATCTCTTGTCCCTTAAGGCAACAGTGTTTTTGCCGTAGTTATTTAAGA**GGTGG**CAGTGTTTC

CAGAGAATTCGTCATGGTCGTCTTGTCGTCTCTTGTTCATCTCTTGTCCCTTAAGGCAACAGTGTTTTTGCCGTAGTTATTTAAGA**GGTGG**CAGTGTTTC

-400 CAGAAGTTTCA**GGCGG**CAGTCTAAGGTAGAAA**TATA**TCCTGTAATATATCCCTCACAGCCGTAATAATACGCGTTGCTGCCACCCCCCACCGCCTTCTG-

CAGAAGTTGGA**GGCGG**CAGTCTAAGGTAGAAA**TATA**TCCTGTAATATATCCCTCACAGCCGTAATAATACGCGTTGCTG-CACCCCCCACCG-CTTCTGC

-300 ---CC-TCC--CTG**CAAA**TTTCTTCTTCAGCTTCCGCTTCTC**CAAG**-TTTCCACCTCGTCCT**TCAACAA**CGACCACCACATCCACA**TCAA**CCA**TCAACAA**

TCCCCTTCCAACTG**CAAA**TTTCTTC-TCAGCTTCCGCTTCTC**CAAG**TTTTCCACCTCGTCCT**TCAACAA**CGACCACCACATCCACA**TCAA**CCA**TCAACAA**

-200 CATCCAACCTCACCATCCATTCGCGTCCTTCCTACCGCCATTACACACTTGGGCCCCCCCCC-CTTTCTCATCGCTAATA**TCAACAG**CCACAACACCCTT

CATCCAACCTCACCATCCATTCGCGTCCTTCCTACCGCCATTACACACTTGGGCCCCCCCCCACTTTCTCATCGCTAATA**TCAACAG**CCACAACACCCTT

-100 TAA*CTCTCCTTTCTCTCCCTCTCCGCTTCTCTCCTTTTTCCC*GCCTATCTG-CCCTACTACTCAGGCTATATCCTCG~~CCTGCTATCGCTTGTAGTTCACA~~

TAA*CTCTCCTTTCTCTCCCTCTCCGCTTCTCTCCTTTTTCCC*GCCTATCTGTCCCTACTACTCAGGCTATATCCTCGCCTGCTATCGCTTGTAGTTCACA

**Figure S2. Alignment of *Pb18* Actin gene promoter region to the corresponding genomic DNA of IVIC *Pb*73**. Light shading denotes identical nucleotides. Identified potential core promoter elements are marked as follow: TATA box elements are dark shaded and transcripti on initiator elements are typed in bold. The R.Y tract element is typed in italic. The corresponding deleted sequence is underlined while Prm_Act_-F/R annealing sequences are cross-lined.
