# Supplementary material for: New Developments of RNAi in Paracoccidioides brasiliensis: Prospects for High-Throughput, Genome-Wide, Functional Genomics
Source: PLoS Negl Trop Dis. 2014 Oct 2;8(10):e3173. doi: 10.1371/journal.pntd.0003173 (PMC4183473; doi:10.1371/journal.pntd.0003173)
Supplement: Figure S3 — Alignment of Pb18 GP43 gene promoter region to the corresponding genomic DNA of IVIC Pb 73. Light shading denotes identical nucleotides. Identified potential core promoter elements are marked as follow: TATA box elements are dark shaded and transcription initiator elements are typed in bold. The R.Y tract element is typed in italic. (DOCX) [file pntd.0003173.s003.docx]

Supporting information: Figure S3.

-100 -80 -60 -40 -20 -1

|.........|.........|.........|.........|.........|.........|.........|.........|.........|........|

*Pbr*B339 -400 CCAGTTGAAAAAATGCGCAT

*Pb18* CCAGTTGAAAAAATGCGCAT

-300 GCCCCACTCAATAAGGGCCTGGTGTGATTCTCATGTTACAGCAAGCCCTGGCATCTGCTGTTGATCTTTTCCTTATTTTGTGGATTTTTGTCCTTTTCAA

GCCCCACTCAATAAGGGCCTGGTGTGATTCTCATGTTACAGCAAGCCCTGGCATCTGCTGTTGATCTTTTCCTTATTTTGTGGATTTTTGGCCTTTTCAA

-200 GGTTGCTTTTGACTGAGAGCTATCACCTGTGGACTCATCTTAAAGCTCACTTGGACCATTCCATGTTTCATTGTTGCAGATTTATCAACAATTATGCACC

GGTTGCTTTTGACTGAGAGCTATCACCTGTGGACTCATCTTAAAGCTCACTTGGACCATTCCATGTTTCATTGTTGCAGATTTATCAACAATTATGCACC

-100 TGCACAGAGGGATTATGGTGTATAAATATCTGCTG*TCTCCTCCCTATTTCCCTCCTCCTTCT*TTTTACAGATCT**TCAAG**GTTTTGGTGGT**CATA**GGCATC

TGCACAGAGGGATTATGGTGTATAAATATCTGCTG*TCTCCTCCCTATTTCCCTCCTCCTTCT*TTTTACAGATCT**TCAAG**GTTTTGGTGGT**CATA**GGCATC

**Figure S3. Alignment of *Pb18* GP43 gene promoter region to the corresponding genomic DNA of IVIC *Pb*73**. Light shading denotes identical nucleotides. Identified potential core promoter elements are marked as follow: TATA box elements are dark shaded and transcription initiator elements are typed in bold. The R.Y tract element is typed in italic.
