# Supplementary material for: New Developments of RNAi in Paracoccidioides brasiliensis: Prospects for High-Throughput, Genome-Wide, Functional Genomics
Source: PLoS Negl Trop Dis. 2014 Oct 2;8(10):e3173. doi: 10.1371/journal.pntd.0003173 (PMC4183473; doi:10.1371/journal.pntd.0003173)
Supplement: Figure S4 — Validation of the Zeocin/ZeoR positive selection system for the genetic manipulation of P. brasiliensis . Pb18 yeast cell colonies (white arrows) expressing the ZeoR selection marker from the “PrmCBP1::Shble::TtrGP43” cassette inserted into genomic DNA by ATMT. (A–E). Light microscope images of viable and stable Pb18 transformants growing on the surface of selective medium at 72 h (3 days), 120 h (5 days) and 168 h (7 days) after the recovery cultivation step; amplified 40×. (F). Representative image of control yeast cells at 168 h (7 days), transformed with an empty pCAMBIA0380 vector, at 168 h of growth in selective medium; amplified 40×. (DOCX) [file pntd.0003173.s004.docx]

Supporting information: Figure S4.


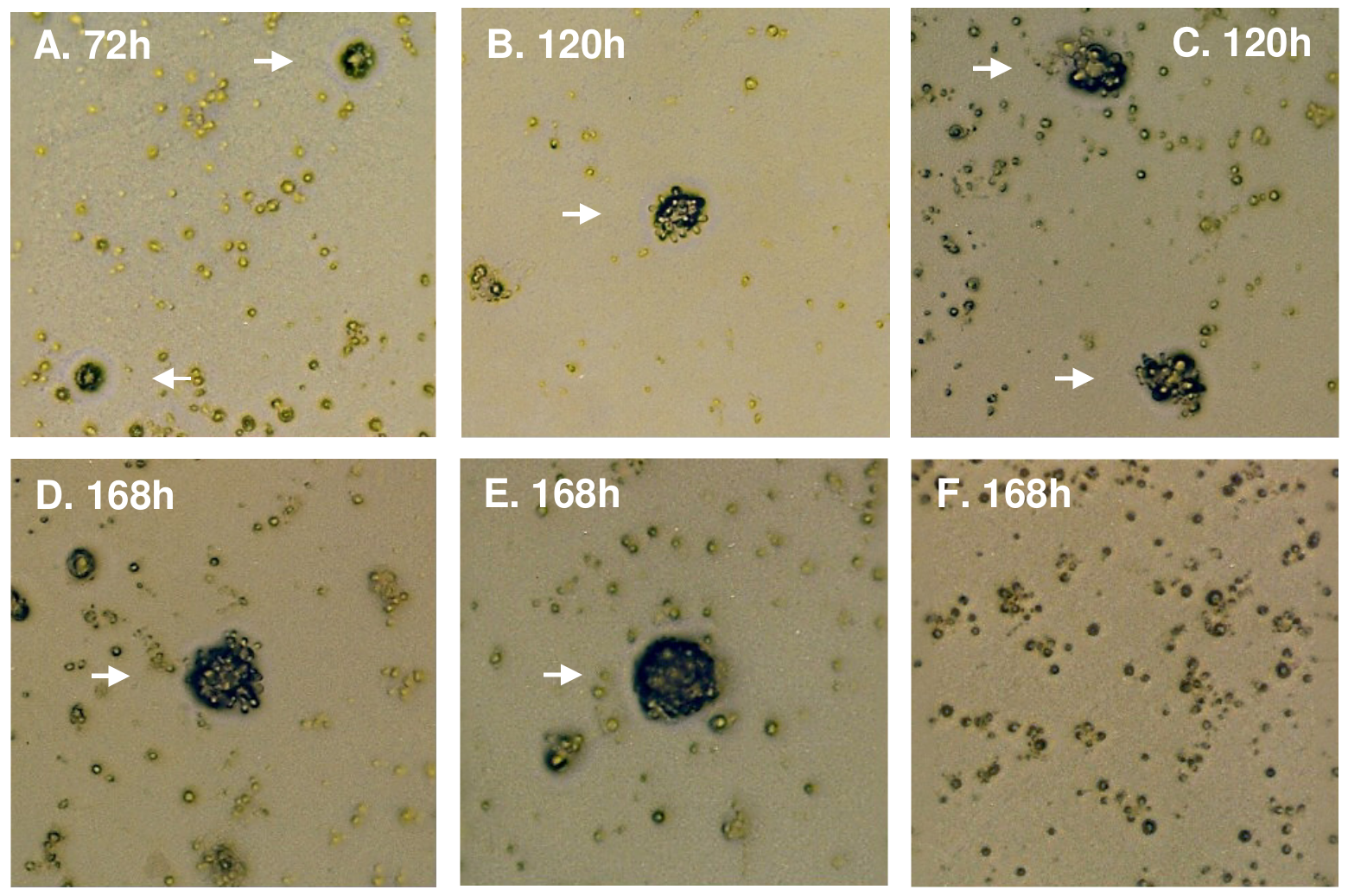


**Figure S4. Validation of the Zeocin/ZeoR positive selection system for the genetic manipulation of *P. brasiliensis.*** *Pb18* yeast cell colonies (white arrows) expressing the ZeoR selection marker from the “Prm_CBP1_::*Shble*::Ttr_GP43_” cassette inserted into genomic DNA by ATMT. (**A-E**). Light microscope images of viable and stable *Pb18* transformants growing on the surface of selective medium at 72h (3 days), 120h (5 days) and 168h (7 days) after the recovery cultivation step; amplified 40X. (**F**). Representative image of control yeast cells at 168h (7 days), transformed with an empty pCAMBIA0380 vector, at 168h of growth in selective medium; amplified 40X.
