# Supplementary material for: New Developments of RNAi in Paracoccidioides brasiliensis: Prospects for High-Throughput, Genome-Wide, Functional Genomics
Source: PLoS Negl Trop Dis. 2014 Oct 2;8(10):e3173. doi: 10.1371/journal.pntd.0003173 (PMC4183473; doi:10.1371/journal.pntd.0003173)
Supplement: Table S1 — Reference set of CDS used to determine codon usage preferences for P. brasiliensis . (DOCX) [file pntd.0003173.s005.docx]

Supporting information: Table S1.

| **Table S1. Reference set of CDS used to determine codon usage preferences for *P. brasiliensis.*** | | | |
| --- | --- | --- | --- |
| CDS | Biological process | GenBank | GC (%) |
| Alcohol dehydrogenase protein | Metabolic process | AY057386 | 52.8 |
| ATP synthase subunit 4 | ATP synthesis | AY271747 | 55.6 |
| Catalase | Response to oxidative stress | AF428076 | 55.9 |
| ClpA heat shock protein | Protein binding | AY229978 | 51.1 |
| ClpB ATP protease | Protein metabolic process | AF449501 | 49.9 |
| Copper-zinc superoxide dismutase | Metabolic process | AY918889 | 54.2 |
| Enolase | Glycolysis | EF558735 | 53.8 |
| Formamidase | Metabolic process | AY163575 | 53.6 |
| Fructose 1,6-biphosphate aldolase 1 | Glycolysis | AY233454 | 53.4 |
| Glyceraldehyde-3-phosphate dehydrogenase | Glycolysis | AY061958 | 55.6 |
| Heat shock protein 60 | Protein binding | AF059523 | 56.5 |
| Heat shock protein 70 | Protein binding | AF386787 | 54.7 |
| Heat shock protein 90 | Protein binding | AY928608 | 52.0 |
| High affinity copper transporter | Ion transport | DQ534496 | 50.0 |
| Manganese superoxide dismutase | Metabolic process | AY905694 | 53.8 |
| Prohibitin | Ion transport | AY603793 | 52.4 |
| Ribosomal protein L10 | Translation | AY204754 | 54.0 |
| Ribosomal protein L35 | Translation | AF416509 | 53.2 |
| Triose phosphate isomerase | Metabolic process | AY250089 | 50.0 |
| Y20 protein | Oxido reductase activity | AF452883 | 56.0 |
| 40S ribosomal protein S12 | Translation | AY190516 | 58.8 |
| 43 KDa secreted glycoprotein precursor | Adhesion | U26160.3 | 51.8 |
