# Supplementary material for: New Developments of RNAi in Paracoccidioides brasiliensis: Prospects for High-Throughput, Genome-Wide, Functional Genomics
Source: PLoS Negl Trop Dis. 2014 Oct 2;8(10):e3173. doi: 10.1371/journal.pntd.0003173 (PMC4183473; doi:10.1371/journal.pntd.0003173)
Supplement: Table S2 — Comparison of synonymous codon frequency in highly expressed CDS. (DOCX) [file pntd.0003173.s006.docx]

Supporting information: Table S2.

| **Table S2. Comparison of synonymous codon frequency in highly expressed CDS.** | | | | | | | | | | |
| --- | --- | --- | --- | --- | --- | --- | --- | --- | --- | --- |
| AA | Codon | *Sce*^a^ | *Ppa*^b^ | *Pbr* |  | AA | Codon | *Sce*^a^ | *Ppa*^b^ | *Pbr* |
| Lys (K) | AAA | 20.7 | 22.1 | 31.3 |  | Glu (E) | GAA | 92.2 | 39.1 | 46.4 |
|  | AAG | 79.3 | 77.9 | 68.7 |  |  | GAG | 7.8 | 60.9 | 53.6 |
| Asn (N) | AAT | 82.5 | 24.4 | 34.9 |  | Asp (D) | GAT | 44.0 | 41.0 | 54.0 |
|  | AAC | 17.5 | 75.6 | 65.1 |  |  | GAC | 56.0 | 59.0 | 45.2 |
| Thr (T) | ACA | 4.7 | 8.8 | 13.6 |  | Ala (A) | GCA | 4.0 | 8.6 | 14.7 |
|  | ACT | 50.0 | 47.5 | 24.6 |  |  | GCT | 67.7 | 59.0 | 29.1 |
|  | ACG | 1.6 | 3.1 | 11.6 |  |  | GCG | 1.0 | 1.2 | 14.2 |
|  | ACC | 43.8 | 40.6 | 50.2 |  |  | GCC | 27.3 | 31.3 | 42.6 |
| Ile (I) | ATA | 3.1 | 2.6 | 4.2 |  | Gly (G) | GGA | 1.5 | 20.4 | 16.9 |
|  | ATT | 46.9 | 52.6 | 36.4 |  |  | GGT | 94.2 | 70.8 | 29.8 |
|  | ATC | 50.0 | 44.8 | 59.4 |  |  | GGG | 0.1 | 2.2 | 10.8 |
| Met (M) | ATG | 100.0 | 100.0 | 100.0 |  |  | GGC | 4.4 | 6.7 | 42.5 |
| His (H) | CAT | 31.6 | 32.2 | 42.5 |  | Val (V) | GTA | 2.5 | 5.0 | 8.6 |
|  | CAC | 68.4 | 67.8 | 57.5 |  |  | GTT | 56.3 | 49.1 | 23.8 |
| Gln (Q) | CAA | 97.1 | 68.0 | 31.7 |  |  | GTG | 3.8 | 9.5 | 21.4 |
|  | CAG | 2.9 | 32.0 | 68.3 |  |  | GTC | 37.5 | 36.5 | 46.2 |
| Pro (P) | CCA | 85.4 | 58.9 | 27.4 |  | Stop | TAA | 75.0 | 59.4 | 32.0 |
|  | CCT | 12.2 | 35.0 | 24.4 |  |  | TAG | 25.0 | 34.4 | 52.0 |
|  | CCG | 0.0 | 1.6 | 16.3 |  |  | TGA | 0.0 | 6.3 | 16.0 |
|  | CCC | 2.4 | 4.5 | 31.9 |  | Tyr (Y) | TAC | 84.9 | 76.5 | 58.4 |
| Arg (R) | AGA | 82.7 | 68.9 | 11.3 |  |  | TAT | 15.2 | 23.5 | 41.6 |
|  | AGG | 1.9 | 3.6 | 9.6 |  | Ser (S) | AGT | 3.0 | 5.6 | 8.9 |
|  | CGA | 0.6 | 0.6 | 10.2 |  |  | AGC | 3.0 | 3.6 | 15.7 |
|  | CGT | 15.4 | 23.4 | 26.9 |  |  | TCA | 6.0 | 9.9 | 8.7 |
|  | CGG | 0.3 | 0.0 | 11.1 |  |  | TCT | 52.2 | 46.1 | 18.7 |
|  | CGC | 1.1 | 1.9 | 31.2 |  |  | TCG | 1.5 | 3.9 | 11.2 |
| Leu (L) | CTA | 9.8 | 5.8 | 5.7 |  |  | TCC | 34.3 | 31.0 | 36.8 |
|  | CTT | 2.4 | 13.7 | 17.8 |  | Cys (C) | TGT | 88.9 | 82.0 | 41.1 |
|  | CTG | 1.2 | 15.7 | 23.5 |  |  | TGC | 11.1 | 18.0 | 58.9 |
|  | CTC | 1.2 | 4.2 | 34.4 |  | Trp (W) | TGG | 100.0 | 100.0 | 100.0 |
|  | TTA | 18.3 | 9.8 | 4.6 |  | Phe (F) | TTT | 23.1 | 29.5 | 29.2 |
|  | TTG | 67.1 | 50.8 | 14.0 |  |  | TTC | 76.9 | 70.5 | 70.8 |

a. Data from [63].

b. Data from [64].

Highly frequent codons are highlighted in gray. *Sce* (*S. cerevisiae*); Ppa (*P. pastoris*); *Pbr* (*P. brasiliensis*).
