# Supplementary material for: New Developments of RNAi in Paracoccidioides brasiliensis: Prospects for High-Throughput, Genome-Wide, Functional Genomics
Source: PLoS Negl Trop Dis. 2014 Oct 2;8(10):e3173. doi: 10.1371/journal.pntd.0003173 (PMC4183473; doi:10.1371/journal.pntd.0003173)
Supplement: Table S3 — Set of oligonucleotides for the in vitro assembly of synthetic codon-optimized mCherry DNA template. (DOCX) [file pntd.0003173.s007.docx]

Supporting information: Table S3.

| **Table S3. Set of oligonucleotides for the *in vitro* assembly of synthetic codon-optimized mCherry DNA template.** | |
| --- | --- |
| Oligo | Sequence (5´ → 3´) |
| 1S | ATGGTCTCCAAGGGTGAAGAAGATAACATGGCTATCATCAAGGAATT**CATGCGTTTCAAGGTCCACA** |
| 3S | **TGAAGGTGAAGGTCGTCCTT**ACGAGGGTACCCAAACCGCTAAGCTCAAGGTCACCAAGGG**TGGTCCTCTCCCTTTCGCTT** |
| 5S | **TTACGTCAAGCACCCTGCTG**ATATCCCTGATTACCTCAAGCTCTCCTTCCCTGAAGGTTT**CAAGTGGGAACGTGTCATGA** |
| 7S | **CTCCCTCCAAGACGGTGAAT**TCATCTATAAGGTCAAGCTCCGTGGTACCAACTTCCCTTC**CGACGGTCCTGTCATGCAGA** |
| 9S | **CCCTGAGGACGGTGCTCTCA**AGGGTGAGATCAAGCAGCGTCTCAAGCTCAAGGACGGTGG**TCACTACGACGCTGAGGTCA** |
| 11S | **TGCTTATAATGTCAATATCA**AGCTCGATATCACCTCCCATAATGAGGATTACACCATCGT**CGAGCAGTACGAGCGTGCTG** |
| 2A | **AAGGACGACCTTCACCTTCA**CCTTCGATCTCGAACTCATGACCGTTGACGGAACCTTCCA**TGTGGACCTTGAAACGCATG** |
| 4A | **CAGCAGGGTGCTTGACGTAA**GCCTTGGAACCATACATGAATTGAGGGGAGAGGATATCCC**AAGCGAAAGGGAGAGGACCA** |
| 6A | **ATTCACCGTCTTGGAGGGAG**GAGTCCTGGGTGACGGTGACGACACCACCATCTTCGAAGT**TCATGACACGTTCCCACTTG** |
| 8A | **TGAGAGCACCGTCCTCAGGG**TACATACGTTCGGAGGAAGCCTCCCAACCCATGGTCTTCT**TCTGCATGACAGGACCGTCG** |
| 10A | **TGATATTGACATTATAAGCA**CCAGGGAGCTGGACAGGCTTCTTAGCCTTATAGGTGGTCT**TGACCTCAGCGTCGTAGTGA** |
| 12A | CTACTTATAGAGCTCATCCATACCACCGGTGGAATGACGACCCT**CAGCACGCTCGTACTGCTCG** |
