# Supplementary material for: New Developments of RNAi in Paracoccidioides brasiliensis: Prospects for High-Throughput, Genome-Wide, Functional Genomics
Source: PLoS Negl Trop Dis. 2014 Oct 2;8(10):e3173. doi: 10.1371/journal.pntd.0003173 (PMC4183473; doi:10.1371/journal.pntd.0003173)
Supplement: Table S4 — Set of oligonucleotides for the construction of expression cassettes. (DOCX) [file pntd.0003173.s008.docx]

Supporting information: Table S4.

| **Table S4. Set of oligonucleotides for the construction of expression cassettes.** | | | |
| --- | --- | --- | --- |
| Segment | Oligo | Restrict site | Sequence (5´ → 3´) |
| Ttr_GP43_ | F | *Pst I* | G**CTGCAG**TGGGACTTTTTACGGCTTGG |
|  | R | *Hind III* | G**AAGCTT**AAGAAAGAAAATCAAATAAAATAACGCCC |
| mCh | F | *Xho I; Sal I* | G**CTCGAGGTCGAC**GAAGGAGATAGTATAATGGTCTCCAAGGGTGAAGA |
|  | R | *Pst I* | G**ctgcag**CTACTTATAGAGCTCATCCATAC |
| *Shble* | F | *Sal I* | G**GTCGAC**ATGGCCAAGTTGACCAGTGC |
|  | R | *Xho I* | C**CTCGAG**GTCCTGCTCCTCGGCCA |
| Prm*_Act_* | F | *BamH I* | G**GGATCC**TCGCCCTTACTATAGGGCACG |
|  | R | *Sal I* | C**GTCGAC**TGTGAACTACAAGCGATAGCAGG |
| Prm_GP43_ | F | *BamH I* | G**GGATCC**CCAGTTGAAAAAATGCGCATGC |
|  | R | *Sal I* | G**GTCGAC**GATGCCTATGACCACCAAAAC |
| Prm_CBP1_ | F | *BamH I* | G**GGATCC**ttatactgatgtctgaacaAtatactag |
|  | R | *Sal I* | G**GTCGAC**TTTGAATGACGAAGTGGTTGTTCT |
| Rfc | F | *Xba I* | G**TCTAGA**ACAAGTTTGTACAAAAAAGCTGAAC |
|  | R | *Pac I* | G**TTAATTAA**ACCACTTTGTACAAGAAAGCTGAA |
| *ccd*B::Cm^R^-invert | F | *Not I* | G**GCGGCCGC**ACTGGCTGTGTATAAGGGAG |
|  | R | *Sal I* | G**GTCGAC**ATTAGGCACCCCAGGCTTTA |
| Intr_GP43_ | F | *Pac I* | G**TTAATTAA**GTGGTAAGTTACATAGTCCC |
|  | R | *Pac I* | C**TTAATTAA**GGACTGGGAGTGAGATATTC |
| GTW_GP43_ | F | **-** | GGGGACAAGTTTGTACAAAAAAGCAGGCTATGAATTTTAGTTCTCTTAACCTGGC |
|  | R | **-** | GGGGACCACTTTGTACAAGAAAGCTGGGTTGACCGCTGTTGTCGATGC |
