# Supplementary material for: New Developments of RNAi in Paracoccidioides brasiliensis: Prospects for High-Throughput, Genome-Wide, Functional Genomics
Source: PLoS Negl Trop Dis. 2014 Oct 2;8(10):e3173. doi: 10.1371/journal.pntd.0003173 (PMC4183473; doi:10.1371/journal.pntd.0003173)
Supplement: Text S1 — Expression cassette assembly. (DOCX) [file pntd.0003173.s009.docx]

Supporting information: Expression cassette assembly.

For the construction of the ihpRNA expression cassette (Figure 1G), the Gateway Rfc (*att*R1::*ccd*B::Cm^R^::*att*R2) was amplified from pET-DEST42 vector with Rfc-F/R primers and cloned, initially, into pCR2.1-TOPO vector. The Rfc was then excised with the pair of enzymes *Xba*I/*Hind*III and cloned into pUC18 vector at their respective restriction sites. Next, pUC18-Rfc was treated with *Not*I/*Sal*I enzymes to remove the *ccd*B and chloramphenicol resistance gene segment (*ccd*B::Cm^R^) from the Rfc. Subsequently, this same segment was amplified from pET-DEST42 vector with *ccd*B::Cm^R^ invert-F/R primers and cloned into pCR2.1-TOPO vector. The “*ccd*B::Cm^R^” was then excised with *Not*I/*Sal*I enzymes and cloned into pUC18-Rfc_Δ_*_ccd_*_B::CmR_, which had been previously treated with the same pair of enzymes, thereby inverting the orientation of this segment in pUC18-Rfc. Afterwards, the “Rfc*_ccd_*_B::CmR-invert_” was excised with *Xho*I/*Sac*I enzymes from pUC18 and cloned into pUC18-Ttr_GP43_ at *Sac*I/*Sal*I sites. Subsequently, the original Rfc was excised from the abovementioned pUC18-Rfc with *Sac*I/*Pac*I enzymes and then cloned into pUC18-Rfc*_ccd_*_B::CmR-invert_::Ttr_GP43_ at their respective restriction sites. In order to confer stability to the inverted repeat constructs in *E. coli* and in *P. brasiliensis* yeast cells, the intron sequence of the GP43 gene (Intr_GP43_) was amplified from *Pb18* genomic DNA with Intr_GP43_-F/R primers and cloned into pUC18-Rfc::Rfc*_ccd_*_B::CmR-invert_::Ttr_GP43_ at *Pac*I site. Finally, the assembled cassette segment “Rfc::Intr_GP43_::Rfc*_ccd_*_B::CmR-invert_::Ttr_GP43_ was excised from pUC18 with *Xho*I/*Hind*III enzymes and cloned into pC0380-Prom_Act_::*Shble*::mCh::Ttr_GP43_, from which the “*Shble*::mCh::Ttr_GP43_” segment had been previously removed with *Sa* I/*Hind*III enzymes. In turn, the cassette “Prom_Act_::*Shble*::mCh::Ttr_GP43_” was excised with *BamH*I/*Spe*I from pC0380 and cloned into pC0380-Prom_Act_::Rfc::Intr_GP43_::Rfc*_ccd_*_B::CmR-invert_::Ttr_GP43_ at *Bgl*II/*Spe*I restriction sites.
